# Graph Enhanced Multi-Modal Network of 4-D Radar-Camera Fusion for Perception in Autonomous Systems

**DOI:** 10.3390/s26144635

**Published:** 2026-07-22

**Authors:** Yuanzhi Deng, Cheng Chi, Jianhao Shen, Yu Han, Shanyin He, Shaolong Chen

**Affiliations:** 1 School of Sino-German Intelligent Manufacturing, Shenzhen City Polytechnic, Shenzhen 518116, China; dengyuanzhi2020@email.szu.edu.cn (Y.D.); hanyu@szcp.edu.cn (Y.H.); heshanyin@szcp.edu.cn (S.H.); 2 Guangdong Engineering Center for Intelligent Sensing and Flexible Manufacturing, Shenzhen City Polytechnic, Shenzhen 518116, China; 3 College of Urban Transportation and Logistics, Shenzhen Technology University, Shenzhen 518118, China; chicheng@sztu.edu.cn (C.C.); shenjianhao@sztu.edu.cn (J.S.)

**Keywords:** object detection, hypergraph, 4-D radar, sensor fusion, autonomous perception

## Abstract

Modern autonomous systems rely on heterogeneous sensing modalities—including vision sensors, millimeter-wave radar, and LiDAR—yet each individual sensor exhibits characteristic failure modes in challenging real-world conditions. While LiDAR-vision co-processing has received extensive attention, the synergistic potential of 4D radar paired with monocular optics remains comparatively unexplored. To fill this gap, we develop a graph-enhanced multi-modal architecture that jointly leverages sparse 4D radar returns and high-resolution camera imagery for scene-level 3D perception. The proposed system is organized around four tightly coupled processing stages: (i) an image-guided point densification scheme (SAA) that augments sparse radar clouds with camera-derived pseudo measurements; (ii) a pose-invariant cross-modal fusion layer that harmonizes enriched radar features with image descriptors and object saliency maps; (iii) a dynamic hypergraph assembly stage that captures higher-order inter-object and cross-sensor dependencies; and (iv) a HyperGCN inference module that regresses 3D bounding parameters and class labels on the resulting relational graph. Integrating the temporal velocity cues native to radar with the rich appearance information from cameras, the system delivers reliable perception under diverse environmental conditions. On the View-of-Delft (VOD) evaluation suite, the proposed model records an mAP of 69.3 and mAOS of 59.8. A systematic ablation further quantifies how geometric invariance—across translation, rotation, and scale transformations—individually affects end-to-end detection fidelity.

## 1. Introduction

The past decade has witnessed remarkable progress in autonomous driving, largely propelled by the deployment of heterogeneous sensing modalities [1]. Cameras, LiDAR, and radar each deliver complementary cues that are indispensable for reliable scene understanding in dynamic environments [2]. Among them, cameras supply dense texture and color information, whereas LiDAR provides accurate range measurements. Nevertheless, individual sensors exhibit inherent weaknesses: cameras degrade under low illumination or adverse weather, and LiDAR struggles with objects of low reflectivity or fast-moving elements [3].

Millimeter-wave (MMW) radar stands out for its weather-proof operation, extended detection range, and ability to measure Doppler velocity. Compared with conventional 3D MMW radar, the emerging 4D radar additionally resolves the elevation dimension (*z* coordinate) alongside the azimuthal position (*x*) and range (*y*), thereby delivering substantially richer spatial information that facilitates 3D localization and pose estimation of targets. Moreover, 4D radar maintains stable performance across diverse and harsh environmental conditions—unlike cameras that rely on visible light and LiDAR that is vulnerable to precipitation and fog—making it a highly attractive modality for safety-critical autonomous navigation.

**Figure 1:**
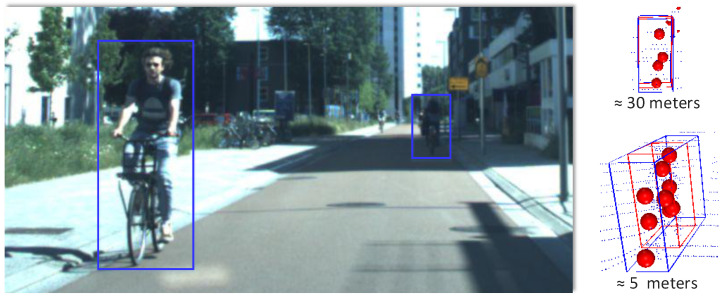
The cyclist can be recognized in camera images but cannot be distinguished from the 4D radar point cloud data. The red blobs represent the 4D MMW radar point clouds, while the blue dots represent LiDAR point clouds.

Notwithstanding these strengths, 4D radar introduces its own difficulties. Chief among them is the characteristically low point density of radar returns, which severely limits the number of scan points per object—a constraint that becomes progressively more pronounced at longer detection ranges. This density deficit frequently produces incomplete object representations, causing fragmented detections and elevated ambiguity, most notably in dense traffic scenes or when targets are partially occluded. As illustrated in Figure 1, relying solely on raw radar returns is generally insufficient for unambiguous object-level recognition.

Prior work has explored the fusion of 3D MMW radar with cameras [4,5,6]; however, the spatio-temporal information embedded in 3D radar remains largely under-exploited for 3D detection. While camera imagery offers high-resolution visual content, it is imperative to devise more effective fusion paradigms that compensate for radar sparsity and bridge the semantic gap between the two modalities so as to achieve higher perceptual accuracy and reliability.

To address the above challenges, this work investigates how 4D radar can be tightly coupled with high-resolution monocular imagery to attain precise, weather-robust scene understanding. The guiding principle is to harness radar’s all-weather sensing capability and Doppler velocity measurements together with the fine-grained visual detail of cameras, thereby overcoming the individual limitations of each modality.

Concretely, we present a graph-enhanced multi-modal neural network that integrates radar and camera streams. The task is scene-level 3D object detection. The architecture is organized into four interconnected processing blocks: a radar point cloud densification module, a pose-invariant cross-modal feature integration module, a hypergraph assembly module, and a HyperGCN specifically designed for 3D detection tasks. The principal contributions are summarized below:

- We devise an image-guided point augmentation mechanism, referred to as the Supervised Attention Augmentation (SAA) block, which increases radar cloud density by synthesizing pseudo returns derived from camera visual guidance.
- We develop a transformation-invariant fusion mechanism that merges densified point clouds and image-derived features for hypergraph construction in a manner invariant to translation, rotation, and scale.
- We design a two-stage HyperGCN dedicated to 3D object detection that extracts hypergraph-structured features from 4D radar point clouds, performing both 3D bounding-box regression and classification. On the VOD benchmark, this model records an mAP of 69.3, demonstrating the effectiveness of the proposed hypergraph-based reasoning under the adopted VOD evaluation protocol.

To clarify the relationship between the proposed modules and existing works, we delineate the original and adapted components as follows. The SAA block shares the high-level idea of using camera detections to guide radar point enrichment with PointPainting [7] and CenterFusion [4]; however, whereas PointPainting appends semantic labels to existing LiDAR points and CenterFusion associates radar detections with image centers via a frustum-based heuristic, our SAA generates physically plausible pseudo-points by random interpolation within image-derived 3D ROIs—a strategy not explored in prior radar-camera fusion work. The transformation-invariant encoding (relative distance *D*, angular offset ϕ, normalized velocity) is inspired by the invariant representation in RadarGNN [8], but we extend it to the multi-modal setting by incorporating heatmap features and hypergraph-structured cross-modal edges, which RadarGNN does not address. The HyperGCN architecture builds upon the hypergraph convolution formulation of HyperGCN [9] and the adaptive construction strategy of AdaHGNN [10]; our original contribution lies in designing a two-stage pipeline (graph embedding followed by hypergraph convolution) specifically tailored for 3D bounding-box regression from radar-camera fusion features, including a detection head and NMS strategy not present in either predecessor. In summary, the SAA densification strategy, the multi-modal invariant fusion with heatmap integration, and the detection-oriented HyperGCN design constitute the principal original contributions of this work.

## 2. Related Work

This section reviews related work associated with 3D object detection methods in perception, focusing on camera-only, LiDAR-only, and fusion-based 3D object detection methods.

### 2.1. Camera-Only 3D Object Detection

YOLO3D [11] was an end-to-end real-time method that outputs the 3D object bounding boxes from a single neural network without any post-processing. It utilized a YOLOv3-based [12] object detection framework but added parameters to output 3D BBox directly. In addition, the YOLO3D employed a PointNet [13] structure to process 3D point cloud data efficiently. Compared with MV3D [14] and AVOD [15], YOLO3D achieved higher effectiveness and real-time performance. Different from YOLO3D, ref. [16] split 3D object detection into two stages: 2D object detection and 3D BBox regression. In the first stage, the rough 2D BBox was generated by the 2D object detector, and the corresponding features were extracted as the region of interest (ROI). In the second stage, these ROI were regressed using a 3D detection network to obtain the accurate 3D bounding box. Subsequently, Monocular3D [17] primarily contributed to generating candidate object proposals. A prior ground-place candidate object location was incorporated into a probabilistic model. Each candidate box projected onto the image plane was scored using several intuitive factors, including semantic segmentation, contextual information, size and location priors, and typical object shapes. However, the pseudo-point cloud generated by Monocular3D was well aligned with the original point cloud in general, but locally it was not aligned accurately enough. Notably, CenterPoint [18] first employed a keypoint-based detector to identify the object’s center and then regressed to other attributes, including 3D size, 3D orientation, and velocity. In the second stage, additional point features on the object were used to improve these estimates. In CenterPoint, 3D object tracking was reduced to greedy nearest-point matching. The resulting detection and tracking algorithms were simple, efficient, and effective.

The image-based methods were promising, but could not provide the precise position and size of the objects compared to LiDAR-based methods.

### 2.2. LiDAR-Only 3D Object Detection

PointPillars [19] introduced a novel encoder that segments the point cloud into clusters of pillars, subsequently processing these pillars. The encoded features were utilized in conjunction with a typical 2D convolutional architecture. The backbone of PointPillars had two sub-networks: a top-down down-sampling network to produce increasingly smaller spatially-resolved features and a second network that performs up-sampling and cascading of features. The output features were used by the detection head to predict the 3D BBox. On the other hand, PointRCNN [20] used PointNet++ [21] functions as the Encoder–Decoder network for feature extraction. It encodes features for each point through four layers of set abstraction, followed by four layers of feature propagation. Segmentation learning for a point is done using annotations from the 3D candidate box. Since background points typically outnumber pre-points, focal loss can be used for category balance during training. These pre-points also assist in generating proposals. While background points are not used for bounding box (BBox) regression, they still contribute to the point feature encoding and decoding due to the network’s receptive field.

Further, 3DSSD [22] proposed a lightweight and efficient point-based 3D single-stage object detector that balances accuracy and efficiency. All essential up-sampling layers and refined operations were removed to reduce computational cost. 3DSSD proposed a new fused sampling strategy in the down-sampling process to make detecting less representative points feasible. 3DSSD outperformed voxel-based single-stage methods and matched two-stage point-based methods in performance. As guided by semantic information, the proposed F-FPS method can exclude much background information and retain more information about the foreground points. However, if only F-FPS is used, many points of the same object will be retained, leading to accuracy degradation. Hence, the authors also consider the sampling information of Euclidean and feature space.

PV-RCNN [23] integrates 3D voxel convolutional neural networks (CNN) with PointNet-based methods to enhance point cloud feature learning. It combines the efficient learning of voxel CNN with the flexible approach of PointNet, using a novel voxel set abstraction module to condense 3D scenes into a small set of key points that capture representative features. By generating high-quality 3D proposals, the model abstracts proposal-specific features from key points to ROI grid points, utilizing keypoint set abstraction with multiple acceptance domains. This approach leads to richer contextual information, improving object confidence and localization compared to traditional pooling.

Unlike the previous, CIA-SSD [24] introduced a single-stage voxel-centric architecture for point-cloud object detection. The backbone shared design similarities with SECOND [25], relying on sparse 3D convolution for efficient volumetric feature extraction. As a single-stage detector, CIA-SSD adopted a structure similar to the Feature Pyramid Network (FPN) [26]. The difference was that CIA-SSD took the mean value of the points in the grid as the starting feature (without adopting the multi-stage multi-layer perceptron (MLP) in VoxelNet [27]), further reduced the computation by continuously decreasing the spatial resolution, and finally spliced the features in the *z* axis to obtain a 2D feature map.

### 2.3. Fusion-Based 3D Object Detection

The camera is susceptible to visual issues such as lighting, weather, shadows, and reflections, leading to inaccurate detection results. The LiDAR typically provides point clouds but lacks color and texture information, making it less accurate when occlusion detection is required. Different environmental conditions and scenarios may require different types of sensors to obtain precise perception data. A single sensor may not be adaptable to all situations, particularly in complex and variable environments. As a result, several representative studies have emerged in sensor fusion for 3D object detection.

MV3D [14] was a fusion-based 3D object detector that seamlessly integrated data from LiDAR and camera. Its workflow involved several key steps: first, it employed a 3D Proposal Network to generate object bounding box proposals from the point cloud bird’s-eye view projections. Next, it independently processed the LiDAR point cloud and camera images, sharing the 3D bounding box proposals to extract features. These proposals were then used to create the ROI in the bird’s-eye view and camera branches. MV3D utilized a region-based fusion network to merge the features from these branches, employing depth-based fusion through per-point averaging. Finally, the fused features were used for object classification and 3D bounding box regression, with each predicted bounding box represented by eight corner points in 3D space. This method enabled the detection of objects in a 3D environment by making the most of LiDAR and camera data fusion. Subsequently, AVOD [15] proposed a specialized 3D object detection method designed for the context of autonomous driving. Its core approach involved two significant steps. Firstly, AVOD aggregated point cloud data from multiple viewpoints, creating various view representations of the scene. These representations aim to comprehensively understand objects in the environment. Secondly, combining the information extracted from the point cloud with 2D image data enabled a more robust and accurate object detection process. By integrating data from point clouds and images, AVOD enhanced the perception capabilities of autonomous driving systems, making it a valuable technique in the field.

Furthermore, F-ConvNet [28] generated 3D frustums based on camera images with the help of 2D ROI. The process involved aggregating point-wise features within each view cone into frustum-level features using PointNet [13]. These features were then arranged into 2D feature maps and passed through a full convolution network for feature extraction. Finally, the detection head was used to achieve end-to-end detection of the directed 3D frame, solving the problem of relying on too few prospective points in the former estimation process.

Notably, PointPainting [7] proposed a new camera–LiDAR fusion strategy. Image semantic segmentation information was projected onto LiDAR points using the LiDAR–camera transformation matrix, before being passed to a baseline object detector. The added semantic cues provide richer object-level guidance and can improve detection accuracy while keeping the model relatively easy to train. In addition, the semantic segmentation output was helpful in 3D object detection, depth estimation, and other autonomous-driving tasks.

In a recent study by Zhou et al. [29], 3D MMW radar and camera sensors were used together for object detection using a new feature-level fusion method. The proposed approach improves feature representation under the bird’s eye view (BEV). The radar points were first temporally accumulated and then sent to a spatio-temporal encoder for radar feature extraction. Simultaneously, 2D features of multi-scale images were obtained from image backbone and neck models, adapted to various spatial scales. The image features were then converted to the BEV using a designed view converter. The study used a two-stage point fusion and ROI fusion to fuse multi-modal features. Finally, the detection head predicted the object category and 3D BBox.

CenterFusion [4] pioneered center-based radar–camera fusion by associating radar detections with image-derived object centers through a frustum-based heuristic, achieving the first competitive radar–camera 3D detector on the nuScenes benchmark. RC-BEVFusion [6] extended this line by introducing a plug-in module that performs radar–camera feature fusion in the bird’s-eye view, leveraging cross-attention to align sparse radar features with dense image BEV features. Both methods, however, rely on 3D radar data without elevation information and employ feature-level or detection-level fusion without graph-structured relational reasoning. In contrast, our approach operates on 4D radar with elevation, employs a graph-based representation that captures higher-order inter-object relationships, and uses a transformation-invariant encoding that is robust to sensor pose changes—design choices that are complementary rather than directly comparable to these BEV-centric methods. We provide a qualitative discussion of these differences in Section 4.

While there have been many 3D object detection perception methods, the main focus has been on using LiDAR and cameras. In addition, due to the sparse point cloud of MMW radar, even if 3D MMW radar is used, it cannot be sufficiently fused with high-resolution images for detection.

Our research proposes a fusion network based on the 4D MMW radar and cameras that densifies the radar point cloud and fuses it in a transformation-invariant manner with camera image features. The proposed HyperGCN takes advantage of the object features in the camera images and establishes the relationship between the sparse point clouds, resulting in effective fusion of image and point cloud features for object classification and 3D BBox regression.

## 3. Methodology

**Figure 2:**
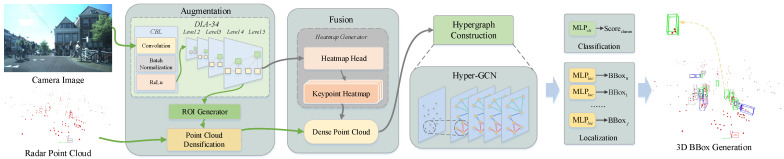
The workflow consists of four main stages: (1) point cloud augmentation, offering a more informative radar point cloud, highlighted in green lines; (2) feature fusion, combining the augmented point cloud data with heatmap features, indicated by grey lines; (3) constructing the hypergraphs that capture intricate relationships and dependencies among various data modalities and objects; (4) HyperGCN then learns from the resulting graph representation by modeling object-to-object and modality-to-modality relationships.

This section details the proposed framework, which is illustrated in Figure 2. The architecture integrates four modules—radar point cloud augmentation, transformation-invariant feature fusion, hypergraph construction, and HyperGCN—into a unified pipeline for 3D object detection in autonomous perception.

The inference procedure proceeds as follows. First, an off-the-shelf detector processes camera images to produce preliminary 3D bounding boxes, which serve as region-of-interest (ROI) proposals. These proposals are then used to identify the associated radar points inside the 4D radar point cloud. The SAA block then enriches these identified returns, producing a denser and more discriminative point representation. Subsequently, the augmented point cloud, together with image features and heatmap responses, is fused in a pose-invariant manner. The fused representations form the basis for constructing a hypergraph, whose nodes and hyperedges encode cross-modal and inter-object relationships. Finally, the HyperGCN consumes the hypergraph and performs 3D object detection, exploiting the high-order relational information captured by the graph structure.

### 3.1. Point Cloud Augmentation

A 4D MMW radar point cloud *P* is defined as an unordered set of points:

(1) $$P=\{P_{i}=\left[x_{i},y_{i},z_{i},rcs_{i},\vec{V_{i}}\right]\in R^{6},\;i=1,\ldots ,N\},$$

where each point is characterized by its absolute 3D coordinates (x,y,z) and radar cross-section rcs. The variable *t* records the frame timestamp and is a global property of the scan rather than a per-point attribute; it is shared across all points within the same frame. The velocity vector $\vec{V}$ adopts dataset-specific conventions. In the VOD dataset [30], $\vec{V}=\left\{v_{r},v_{c}\right\}$, with $v_{r}$ and $v_{c}$ denoting the relative and absolute radial velocities, respectively. In nuScenes [31], the velocity is instead parameterized as $\left(v_{x},v_{y}\right)$.

**Figure 3:**
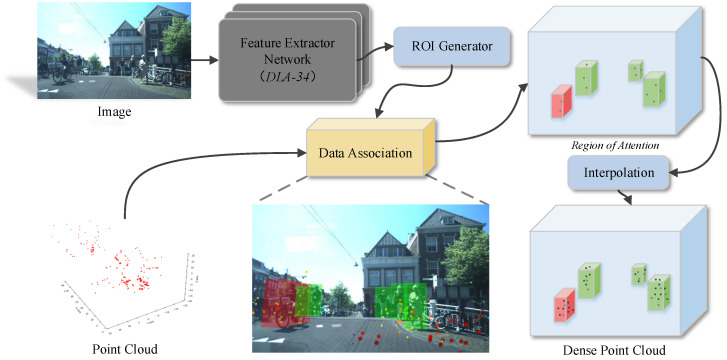
Overview of the radar point cloud densification pipeline. The process of dense point cloud generation involves the following steps: (1) extracting features from RGB camera images and regressing the initial bounding box of the object; (2) mapping these bounding boxes into the 4D radar point cloud and associating them with radar points to obtain the region of attention; (3) conducting random interpolation to augment data density within the region of attention, generating pseudo-points and producing a locally denser point cloud.

Figure 3 illustrates the augmentation pipeline. In the first stage, RGB images are fed into a DLA-34 [32] backbone (following the architecture of [33]) to extract visual features.

In the second stage, the detected bounding boxes are associated with 4D radar points to form 3D ROIs. Concretely, a 3D ROI generator processes the image, and the resulting 2D detections are projected onto the radar point cloud. The association between 2D image detections and 3D radar points is established through a projective transformation chain. In the VOD dataset, the camera calibration provides the intrinsic parameter matrix *p* and the rectification matrix $R_{0}$; the radar-to-camera transformation is performed via $T_{radar}^{cam}$, which maps radar coordinates to the camera coordinate frame. A 3D radar point $X_{radar}={\left[x,y,z,1\right]}^{⊤}$ in homogeneous coordinates is first transformed to the camera frame by $T_{radar}^{cam}$, rectified by $R_{0}$, and then projected onto the image plane by the intrinsic matrix *p*, yielding the pixel coordinate $u={\left[u,v,1\right]}^{⊤}$. The combined transformation matrix $T\in R^{4\times 4}$ for camera *i* is formulated as:

(2) $$u_{i}=p_{i}\cdot R_{0}\cdot T_{radar}^{cam}\cdot X_{radar}=T_{i}\cdot X_{radar},$$

where i∈{1,2} indexes the left and right cameras. Here, $T_{i}=p_{i}\cdot R_{0}\cdot T_{radar}^{cam}$ is the composite projective transformation that maps 3D radar coordinates to 2D image pixels. This projection yields the 3D ROI within the point cloud. However, the associated points often remain insufficient for reliable detection. To remedy this, we apply random interpolation inside each ROI, generating supplementary pseudo points that increase data density and enrich feature representations.

**Figure 4:**
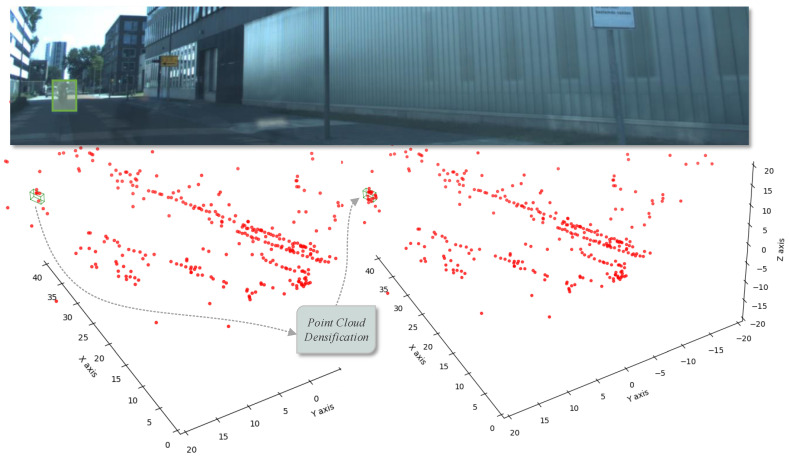
The green bounding box indicates the object in the camera and the corresponding 4D radar point cloud, where the subplot in the bottom right is the augmented point cloud.

Algorithm 1 formalizes the augmentation procedure, where $P_{a}$ denotes the radar points falling inside a 3D ROI and *k* denotes the number of pseudo-points sampled for each ROI. Because the nuScenes radar does not report object height, we set the average height Γ to 1.5 m by convention. The augmented point cloud is denoted $P_{a}+\Delta P$. Figure 4 provides a qualitative visualization of the augmented point cloud.
**Algorithm 1:** Point Cloud Augmentation
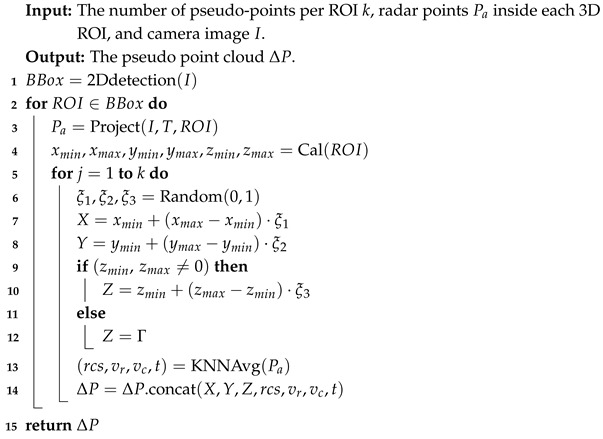


### 3.2. Transformation-Invariant Feature Fusion

In convolutional neural networks, transformation invariance generally refers to the network’s ability to produce consistent outputs under transformations such as translation, rotation, and scaling of the input.

For graph neural networks (GNNs), however, invariance takes a different form because the data are graph-structured. Specifically, GNN invariance concerns the stability of the network’s representations when the arrangement or connectivity of graph nodes varies. This property is critical in perception systems, where the graph topology may evolve across time steps, locations, or scenarios. An essential property of GNNs is their invariance—or partial invariance—to perturbations in node ordering and edge connectivity, which is crucial for applications relying on dynamic graph data [8].

During training of a non-invariant GNN, each ground-truth 3D BBox is represented as (x,y,z,w,l,h,θ), where (x,y,z) are the absolute center coordinates, (h,w,l) are the 3D dimensions (in meters), and θ is the yaw angle. Following Drost et al. [34] and Fent et al. [8], we adopt a relative representation that discards absolute coordinates in favor of positions encoded with respect to neighboring points. Specifically, the offsets dx, dy, and dz between a point and its neighbor are stored in the edge features, preserving spatial relationships while ensuring translation invariance. The resulting representation takes the form (D,ϕ,w,l,h,θ). Instead of absolute coordinates (x,y,z), we use quantities derived from a reference point $p_{0}$ and its nearest neighbor $p_{n}$: *D* denotes the Euclidean distance from $p_{0}$ to the BBox center *O*, and ϕ represents the angular separation between $\vec{p_{0}p_{n}}$ and $\vec{p_{0}O}$. The point $p_{0}$ is sampled uniformly within the ground-truth box, and $p_{n}$ is retrieved via k-nearest neighbors.

**Figure 5:**
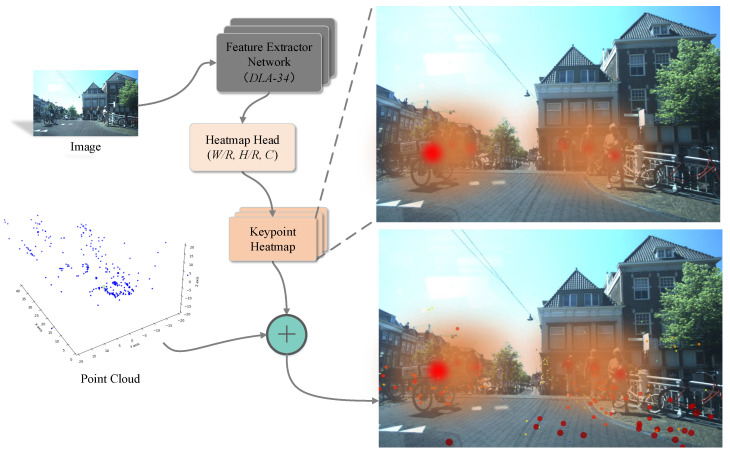
The workflow is divided into two parts: (1) heatmap generation, obtaining the heatmap of the keypoint from the heatmap head; (2) fusing the heatmap with the densified radar point cloud.

Additionally, we extract a heatmap from the camera image, which is later fused with the 4D radar point features. The heatmap generation and fusion workflow is depicted in Figure 5. We adopt the term “heatmap” following the convention established in the keypoint-based detection literature [33,35], where it denotes a spatial probability map indicating the likely location of object centers. While the reviewer correctly notes that a visible-range camera captures reflected sunlight rather than thermal radiation, we use “heatmap” strictly as a computational artifact—a 2D Gaussian response map generated by the detection head—not as a physical thermal image. This terminology is standard in the object detection community and is not intended to imply infrared sensing.

During heatmap training, the ground-truth keypoint $p_{key}$ corresponds to the projected center of each 3D BBox in the image. For every keypoint, we compute its low-resolution counterpart $\tilde{p}=\left\lfloor \frac{p_{key}}{R}\right\rfloor$ and project $p_{key}$ onto the heatmap via a Gaussian kernel:

(3) $$Y_{xyc}=\exp\;\left(-\frac{{\left(x-{\tilde{p}}_{x}\right)}^{2}+{\left(y-{\tilde{p}}_{y}\right)}^{2}}{2\;\sigma_{p_{key}}^{2}}\right).$$

The kernel bandwidth $\sigma_{p_{key}}$ is object-size adaptive [35]. The resulting heatmap $Y\in {\left[0,1\right]}^{\frac{W}{R}\times \frac{H}{R}\times C}$ uses an output stride of R=4 during training, where *C* denotes the number of object categories. When two Gaussian distributions of the same class overlap, the element-wise maximum is retained. Background is encoded as $Y_{xyc}=0$.

Because the discretization from the original resolution to the feature-map resolution introduces quantization error, an offset prediction branch is added to compensate:

(4) $$L_{off}=\frac{1}{N}\sum_{p_{key}}\left|{\tilde{O}}_{p_{key}}-\left(\frac{p_{key}}{R}-\left\lfloor \frac{p_{key}}{R}\right\rfloor \right)\right|,$$

where ${\tilde{O}}_{p_{key}}$ is the predicted offset. The training objective drives ${\tilde{O}}_{p_{key}}$ toward the fractional part of $\left(\frac{p_{key}}{R}-\left\lfloor \frac{p_{key}}{R}\right\rfloor \right)$.

### 3.3. Hypergraph Construction

Hypergraphs are well suited for multi-modal data integration because their nodes and edges can represent heterogeneous data types while simultaneously capturing higher-order relationships among them [36]. In object detection, the inter-object relationships can be highly complex, especially in cluttered scenes. The flexible topology of hypergraphs enables them to model many-to-many associations and dependencies more effectively than standard graphs [37,38].

In our framework, the hypergraph serves as a unified structure that integrates camera and radar information, thereby providing a holistic scene representation for autonomous navigation. Formally, a hypergraph is defined by its node set *V* (|V|=N), edge set *E* (|E|=M), and hyperedge set *H* (|H|=K). Each node $v_{i}\in V$ carries a feature vector $x_{i}\in R^{d}$, each edge $e_{j}\in E$ is associated with a feature $x_{j}\in R^{n}$, and each hyperedge $h_{k}\in H$ has a feature $x_{k}\in R^{q}$.

**Figure 6:**
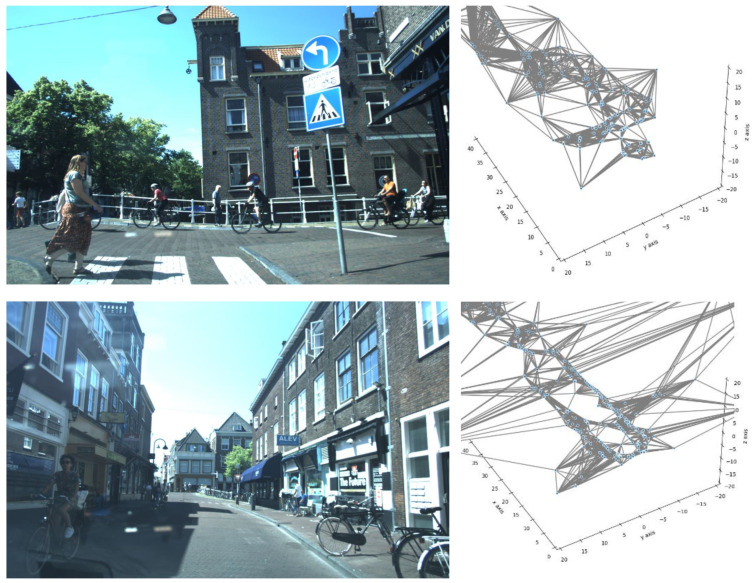
The left column subplot is the camera image and the right column is the visualization of the constructed hypergraph in the 4D radar coordinate system.

The connectivity pattern is encoded via a binary incidence matrix $A\in {\left\{0,1\right\}}^{N\times M}$, where $A_{im}=1$ indicates that node $v_{i}$ belongs to hyperedge $h_{m}$. Each hyperedge is built from the points assigned to the same image-derived 3D ROI proposal, so the graph topology is determined by proposal–point association rather than by ground-truth object labels at inference time. During training, ground-truth boxes supervise the ROI generator and provide classification score for the detection loss; during inference, predicted ROIs and their confidence scores are used to assemble the hyperedges. This design reinforces intra-object coherence while avoiding label leakage in graph construction. Figure 6 visualizes the constructed hypergraphs in the 4D radar coordinate system alongside the corresponding camera images.

Edge features are designed to reduce coordinate dependence: instead of storing absolute positions, they encode relative displacements dx,dy,dz between neighboring points. These relative vectors are translation-invariant and rotation-equivariant, while their derived scalar descriptors (e.g., distances and angles) are invariant to rigid coordinate transformations [8].

We provide a formal justification for the rigid-motion invariance of the scalar feature representation. Let $T:p\mapsto p^{\prime }$ denote a rigid transformation composed of a rotation R∈SO(3) and a translation $t\in R^{3}$, such that $p^{\prime }=Rp+t$ for every 3D point p.

Translation invariance. For two points $p_{0}$ and $p_{n}$, the relative displacement $\Delta p_{0n}=p_{n}-p_{0}$ eliminates the translation term: $\Delta p_{0n}^{\prime }=\left(Rp_{n}+t\right)-\left(Rp_{0}+t\right)=R\Delta p_{0n}$. Thus, the vector is unaffected by translation and changes only by rotation. The same cancellation holds for the offset from $p_{0}$ to the box center O, $\Delta p_{0O}=O-p_{0}$. Consequently, scalar quantities derived from these offsets, such as $D_{0n}=∥\Delta p_{0n}∥$, $D_{0O}=∥\Delta p_{0O}∥$, and the angle ϕ between them, are invariant to any global translation.

Rotation invariance. Under rotation *R*, both $\Delta p_{0n}$ and $\Delta p_{0O}$ are transformed by the same orthogonal matrix. Since $R^{T}R=I$, dot products and Euclidean norms are preserved, so the angular descriptor remains unchanged:

(5) $$\cos\phi^{\prime }=\frac{{\left(R\Delta p_{0n}\right)}^{T}\left(R\Delta p_{0O}\right)}{∥R\Delta p_{0n}∥\;∥R\Delta p_{0O}∥}=\frac{\Delta p_{0n}^{T}\Delta p_{0O}}{∥\Delta p_{0n}∥\;∥\Delta p_{0O}∥}=\cos\phi .$$

The velocity magnitude $∥\vec{V}∥$ is likewise invariant under rotation of the coordinate frame, assuming the velocity vector is rotated consistently with the points. The radar cross section rcs is treated as a scalar radar attribute, and the heatmap feature *Y* is an image-derived confidence value attached to each node. Therefore, the scalar node representation $\left(D_{0O},\phi ,rcs,∥\vec{V}∥,Y\right)$ is invariant to global rigid coordinate transformations of the 3D radar frame.

Scale behavior. The angular term ϕ and normalized velocity direction are dimensionless, whereas distance terms such as $D_{0O}$ scale proportionally under a uniform coordinate scaling. Hence, the representation is strictly invariant to rigid motions (translation and rotation), while scale robustness requires fixed metric units or explicit distance normalization. In our implementation, all datasets are processed in metric coordinates, so scale changes are not introduced as arbitrary coordinate transformations.

To achieve translation invariance, absolute coordinates are excluded from the feature set, and structural information is derived from relative geometry and edge connectivity. Rotation invariance of the scalar geometric descriptors follows from using distances, angles, and velocity magnitudes rather than absolute coordinate directions. Note that nuScenes provides only 2D velocity components ($\vec{V}=\left(v_{x},v_{y}\right)$) because its 3D radar lacks elevation measurements, whereas the VOD 4D radar measures radial velocities including the *z* component ($\vec{V}=\left(v_{x},v_{y},v_{z}\right)$).

By combining the transformation-invariant features with the heatmap, each node in the hypergraph is represented by a feature vector:

(6) $$x_{i}=\left[D_{i},\;\phi_{i},\;\vec{V},\;rcs,\;t,\;Y\right],$$

where *Y* denotes the heatmap feature, and $D_{i}$ and $\phi_{i}$ are the transformation-invariant geometric descriptors (Euclidean distance and angular offset, as defined in Section 3) derived from the radar point coordinates. These relative geometric features encode the spatial layout of radar returns without exposing absolute coordinates, thereby preserving transformation invariance while incorporating radar-derived positional information. The resulting $x_{i}$ encompasses general, heatmap, and transformation-invariant attributes, and the fusion is accomplished by concatenating these heterogeneous features into a unified node representation.

The inclusion of Doppler velocity in the node features provides a discriminative cue that is unavailable from camera or LiDAR alone. The radial velocity $v_{r}$ directly encodes the relative motion between the ego-vehicle and the target, enabling the model to distinguish moving objects (cars, cyclists, pedestrians) from static clutter (guardrails, parked vehicles, infrastructure). This motion-based discrimination is particularly valuable in urban scenes where geometric appearance alone is ambiguous: a parked car and a moving car may produce similar point clouds, but their Doppler signatures differ significantly. Empirically, the ablation confirms that adding the transformation-invariant velocity encoding raises mAP from 64.2 to 69.3 (+5.1 points), demonstrating that velocity is not merely an auxiliary feature but a primary contributor to detection accuracy in the radar-camera fusion setting.

The graph *G* is converted into a matrix representation suitable for neural network processing: an adjacency matrix *A*, a node feature matrix *X*, an edge feature matrix *E*, and a hyperedge incidence matrix *H*. The tuple (A,X,E,H) constitutes the input to our HyperGCN.

### 3.4. Hypergraph Neural Network

To enhance both local feature interaction and global connectivity within the data, we leverage graph convolutional networks (GCNs), which can model complex relationships in non-Euclidean domains and have proven effective for point cloud understanding [39].

Since our densification is limited to camera-visible regions, the hypergraph for HyperGCN is constructed under image guidance rather than as a fully connected graph. The HyperGCN architecture comprises two stages: graph feature embedding and graph convolution.

Graph feature embedding seeks low-dimensional vector representations of nodes. Following Fent et al. [8], we first lift the low-dimensional node and edge features into a high-dimensional space via shared MLPs. Node features are embedded through a four-layer MLP, and edge features through a three-layer MLP. The embedded features are then aggregated (denoted ⊕) to form representations suitable for downstream graph learning. Let ϕ denote the embedding function and $N_{\nu }$ the set of neighbors of node ν. The aggregated node feature is:

(7) $$h_{\nu }^{l+1}=\phi \;\left(h_{\nu }^{l},\;\underset{u\in N_{\nu }}{⨁}\xi \;\left(h_{\nu }^{l},\;h_{u}^{l},\;e_{\nu ,u}^{l}\right)\right).$$

**Figure 7:**
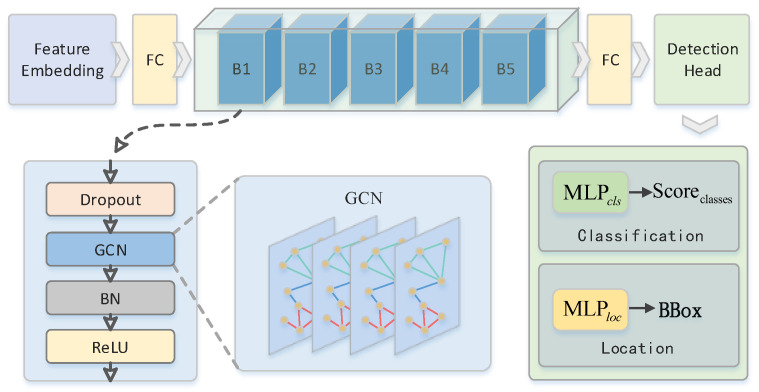
The model structure of HyperGCN consists of two fully connected (FC) layers, five graph convolutional blocks $B_{i}$, and the detection head. Each convolutional block $B_{i}$ has four components: the Dropout layer, the GCN, the batch normalization (BN) layer, and the ReLU activation layer. Before the graph embedding fusion features are propagated to the convolutional block, a fully connected layer is applied to the input features to obtain a low-dimensional representation of the features. Next, the correlation between the set of nodes is established by constructing a hypergraph *G*, overcoming the problem of sparse intrinsic connections between radar points. Each node in the hypergraph represents an element in the data. *X* serves as the feature matrix of the node. *H* is the hypergraph grid, which represents the relationships between nodes.

HyperGCN generalizes standard GCN to the hypergraph domain, enabling the modeling of higher-order relationships [9]. Its architecture is illustrated in Figure 7.

Let *G* denote the hypergraph, *X* the node feature matrix, *H* the hyperedge incidence matrix, *A* the adjacency matrix, and *Y* the heatmap feature. The hypergraph convolution is defined as:

(8) $$G^{\left(l+1\right)}=\sigma \;\left(D_{G}^{-\frac{1}{2}}HAH^{⊤}D_{G}^{-\frac{1}{2}}G^{\left(l\right)}W^{\left(l\right)},\;\underset{u\in N_{\nu }}{⨁}Y\right),$$

where $G^{l}$ are the node features at layer *l*, $W^{l}$ denotes a trainable projection matrix, σ is a non-linear activation, and $D_{G}$ is the degree matrix derived from the hyperedge structure.

## 4. Experiments

We evaluate the proposed framework on two public benchmarks—VOD and nuScenes—covering multiple object categories under both qualitative and quantitative protocols. The experiments demonstrate the effectiveness and cross-dataset generalizability of our approach.

### 4.1. Datasets

VOD. The View-of-Delft (VOD) dataset [30] is a widely adopted automotive benchmark comprising 8693 time-synchronized and calibrated frames from a 64-line LiDAR, a stereo camera pair, and a 3 + 1D MMW radar, all collected in complex urban traffic. It contains 123,106 annotated 3D bounding boxes spanning 26,587 pedestrians, 10,800 cyclists, and 26,949 cars.

The 4D MMW radar in VOD measures distance, speed, height, and azimuth with greater accuracy and resolution than standard 3D radars. The sensor coordinate convention and data format are compatible with the KITTI dataset [40]. The official split partitions the data into training (59%), validation (15%), and testing (26%) subsets. Following the VOD protocol, we adopt two evaluation metrics: Average Precision (AP) and Average Orientation Similarity (AOS). AP is computed from the 3D intersection-over-union (IoU) of predicted and ground-truth boxes, requiring 50% overlap for cars and 25% for pedestrians and cyclists. The class-averaged scores yield mAP and mAOS.

nuScenes. The nuScenes dataset [31] encompasses 1000 driving episodes (each approximately 20 s) recorded in Boston and Singapore, covering urban, suburban, and highway scenarios under diverse weather conditions and times of day. Each episode is equipped with a synchronized sensor suite of six cameras, one LiDAR, five 3D MMW radars, GPS, and IMU, providing a rich resource for autonomous driving research. The dataset contains 28,130 training frames, 6019 validation frames, and 6008 test frames, with annotations for ten object categories: cars, buses, trailers, construction vehicles, trucks, motorcycles, bicycles, pedestrians, barriers, and traffic cones.

Complementing nuScenes, the nuImages extension provides 93,000 multi-category images with 2D annotations captured under varying environmental conditions (rain, snow, nighttime), further enriching the training data and bolstering model robustness.

### 4.2. Implementation Details

#### 4.2.1. Data Processing

The data processing pipeline consists of three stages. First, radar points associated with detected objects are densified. Second, the densified points are fused with the corresponding camera-derived heatmaps. Third, the 7-dimensional raw node features are transformed into the invariant fusion representation $\left(\vec{V},rcs,t,heatmap\right)$ for subsequent graph construction.

The heatmap generator produces approximate 3D ROIs from camera images. A two-step training strategy is adopted for accurate 3D bounding box prediction. To avoid evaluation leakage, model parameters are learned from the training splits, while validation splits are used only for model selection and hyperparameter tuning. Because the backbone was originally trained on the COCO dataset [41], whose category set differs from that of VOD and nuScenes, a two-stage fine-tuning procedure is applied: the COCO-pretrained model is first fine-tuned on nuImages and then further adapted to the target camera images. This yields a detector tailored to the target class vocabularies—VOD focuses on pedestrians, cars, and cyclists, whereas nuScenes encompasses a broader set including buses, trailers, construction vehicles, trucks, motorcycles, bicycles, barriers, and traffic cones.

For data augmentation, each ground-truth 3D BBox is perturbed by a random translation ($T_{x}\in \left(0,2\;m\right),\;T_{y}\in \left(0,2\;m\right),\;T_{z}\in \left(0,2\;m\right)$) and a random rotation around the *z* axis within [−π/4,π/4]. Each point cloud and box is mirrored about the *x* axis with 50% probability. To avoid truncating objects, selection boxes are enlarged by 10%. A proposal is labeled as a positive sample if its IoU with the ground truth exceeds 50%.

**Figure 8:**
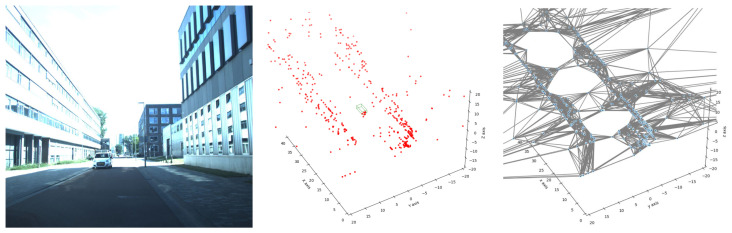
The columns show a VOD camera image, 4D millimeter-wave radar point cloud, and a 3D visualization of the corresponding constructed hypergraph.

After training the 3D ROI generation model, points within the predicted boxes are augmented via Algorithm 1. Figure 8 visualizes the constructed hypergraph from the camera’s front view. The supervised densification strategy confines interpolation to image-derived 3D ROIs, avoiding unnecessary densification of the entire point cloud and thus improving computational efficiency. For objects without any radar returns, pseudo-radar points are created at the 3D ROI locations, with their $\left(\vec{V},rcs,t\right)$ attributes set to the averages of k-nearest neighbors.

#### 4.2.2. Model Details

The 3D ROI model follows the architecture of Duan et al. [33] and is optimized with the ADAM algorithm. No data augmentation is applied during ROI generator training because cropping or scaling would distort the 3D measurements. Image features are extracted using a DLA-34 backbone pretrained on ImageNet [42], with randomly initialized up-sampling layers. ADAM momentum is set to 0.9, the learning rate is initialized at $5\times 10^{-4}$ and halved whenever the validation loss stagnates, the batch size is 32, and training proceeds for 100 epochs. Input images are zero-padded to 1280×384 during both training and inference.

The HyperGCN processes radar–camera fusion features. After transformation-invariant encoding, the raw radar point features are combined with the heatmap to form the hypergraph-structured input. The input and output channel dimensions are 4 and 1024, respectively. A detection head regresses the 3D bounding boxes. Non-maximum suppression (NMS) is employed to merge overlapping predictions; following Shi and Rajkumar [43], the median position and size of overlapping boxes are retained, and the confidence score is computed as the classification score weighted by IoU and occlusion factor.

The HyperGCN framework captures higher-order feature interactions through hyperedge construction and hypergraph convolution. The architecture is inspired by AdaHGNN [10], an adaptive hypergraph-based multi-layer network designed for tag-related feature correlation and higher-order semantic exploration. L2 regularization [44] is applied to mitigate overfitting. Multi-scale node features undergo classification via fully connected layers that reduce dimensions from 5120 to 2048 and then 2048 to 1024. Training uses an end-to-end scheme with a batch size of 4. The output dimensions at different stages are $d_{1}=2048$, $d_{2}=2048$, $d_{3}=1024$, and $d_{4}=1024$. For the car category, the learning rate is initialized at 0.125 and decayed by 0.1 every 400 K steps over 1400 K total steps. For pedestrians and cyclists, a learning rate of 0.32 is used with a decay rate of 0.25 every 400 K steps.

### 4.3. Results

This section reports the performance of our method on the VOD and nuScenes benchmarks and compares it with representative baselines.

#### 4.3.1. VOD

We evaluate 3D object detection on the VOD benchmark, which is a representative 4-D MMW radar dataset. Following the VOD evaluation protocol, detection is performed within the safety-critical “Driving Corridor” for three object categories: car, pedestrian, and cyclist.

Although PointPillars and PointRCNN were originally designed for LiDAR input, they are widely adopted in perception systems. Since no 4D radar-based method exists in the VOD benchmark, we treat the radar point cloud as a sparse LiDAR surrogate for PointPillars. PointRCNN, a two-stage detector, first segments foreground points via PointNet++ [21] to generate 3D proposals, then refines them with a second-stage bounding-box regressor. We also include MonoRCNN++ [45], which leverages a joint probability distribution of object height priors for monocular 3D detection, despite its performance gap relative to LiDAR-based methods. We acknowledge that using LiDAR-designed baselines with sparse radar input may not fully reflect their original capability. To mitigate this concern, we adopt the following fairness safeguards: (i) all baselines and our method use the same VOD training/validation/test split; (ii) identical data augmentation (random translation, rotation, and mirroring) is applied to all radar-based variants; (iii) the same multi-frame aggregation settings (1-scan, 3-scans, 5-scans) are used for both PointPillars and our method; and (iv) radar-specific features (rcs, $v_{r}$) are provided to all baselines whenever the architecture supports them, rather than using LiDAR features alone. Direct comparison with CenterFusion [4] and RC-BEVFusion [6] on VOD is not feasible because both methods require 3D radar data without elevation (nuScenes format) and their code is not publicly released for VOD adaptation. We therefore provide a qualitative comparison: CenterFusion relies on frustum-based radar–camera association without densification, whereas our SAA actively enriches the radar cloud; RC-BEVFusion performs BEV-level feature alignment, while our method models higher-order relationships via hypergraph convolution. These architectural differences make the methods complementary rather than directly comparable on the same benchmark.

We compare multiple sensor–feature combinations. LiDAR features comprise (x,y,z) and reflection intensity, while radar features include spatial coordinates, rcs, and radial velocity $V_{r}$. As shown in Table 1, the LiDAR-only PointPillars model outperforms its radar-only counterpart, confirming that point cloud density is a decisive factor. Notably, the radar-only PointPillars model that incorporates $v_{r}$ achieves a respectable 63.0 mAP and 56.8 mAOS. Among PointRCNN and IA-SSD [46], LiDAR-based variants again lead, further underscoring the importance of point density.

**Figure 9:**
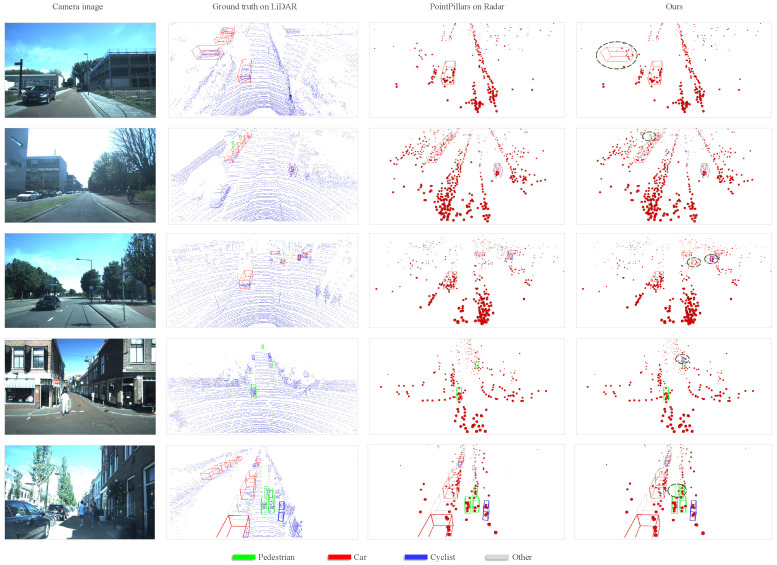
The visual comparison with PointPillars in VOD. The LiDAR point cloud is blue, and the 4D radar points are red. The first column shows RGB images. The second column provides the ground truth on LiDAR. The third column shows detection results on radar-only PointPillars. The fourth column shows the results of our radar–camera fusion method. The dotted circles highlight objects missed by PointPillars but detected by our method.

On the radar side, our method surpasses PointPillars after applying point cloud densification and heatmap fusion to node features (mAP: 60.2 vs. 52.3; mAOS: 48.3 vs. 41.0). When radial velocity $\vec{V}$ is included, our approach reaches an mAP of 69.3 and an mAOS of 59.8, exceeding the radar-only baselines considered in this study. These results indicate that (i) densifying sparse radar points consistently improves detection, (ii) incorporating $\vec{V}$ and heatmap features yields further gains, and (iii) camera–radar fusion is particularly beneficial for sparse radar point clouds in autonomous perception. A qualitative comparison with PointPillars is provided in Figure 9.

#### 4.3.2. nuScenes

To assess the cross-dataset generalizability of our approach, we additionally train and evaluate on nuScenes. The official evaluation metrics include Average Precision (AP), Average Translation Error (ATE), Average Orientation Error (AOE), Average Scale Error (ASE), and Average Velocity Error (AVE). As reported in Table 2, our method obtains the strongest results among the listed radar-based detectors, achieving 13.2% AP for cars and 5.6% AP for trucks and yielding lower errors across ATE, AOE, ASE, and AVE than R-PointGNN [47] and PointNet [13].

It is noteworthy that the overall detection performance on nuScenes remains limited. Many object categories fail to exceed the 10% recall threshold required for AP computation, which poses practical challenges.

The sparsity of 3D radar point clouds in nuScenes means that many camera-visible objects receive few or no radar returns. We therefore compute a performance boundary based on the proportion of annotated objects that generate radar detections. A significant fraction of annotated objects produce no radar points because their reflective properties are insufficient for radar detection. Furthermore, the nuScenes 3D radar does not provide height information, making it difficult for the model to meet the 10% recall threshold. These inherent data limitations constrain the achievable detection performance.

We position the nuScenes evaluation as an auxiliary validation of cross-dataset generalization rather than a strong proof of detection performance, for the following reasons. First, class imbalance is severe: the nuScenes training set contains far more car and truck instances than pedestrians or cyclists, biasing the model toward dominant categories. Second, the absence of radar height information (*z* coordinate) in nuScenes 3D radar prevents our SAA from generating elevation-aware pseudo-points, degrading the quality of densified clouds. Third, failure case analysis reveals that missed detections predominantly occur for (i) distant objects (>40 m) where radar returns are extremely sparse, (ii) small objects (pedestrians, traffic cones) whose radar cross-section falls below the detection threshold, and (iii) partially occluded objects where the camera ROI is incomplete. These limitations are inherent to the sensor configuration of nuScenes and do not indicate a fundamental deficiency of the proposed architecture, which achieves substantially stronger results on the 4D radar VOD benchmark where elevation and higher-density returns are available.

### 4.4. Ablation Studies

We conduct ablation experiments on the VOD dataset within the “Driving Corridor” region [30] to quantify the contribution of each design choice to the overall detection performance.

#### 4.4.1. Effect of Radar Multi-Scan Aggregation

This experiment examines how aggregating multiple radar scan frames affects detection quality. Temporal aggregation can increase point density but also introduces additional noise [48]. We train and evaluate both PointPillars and our network using the last three and five radar scans (3-scans and 5-scans). Table 3 confirms that both models benefit from richer multi-scan data. PointPillars improves from 63.0 to 69.4 mAP with 3-scans and reaches 70.8 mAP with 5-scans. Our method consistently outperforms PointPillars across all multi-scan settings, achieving 73.9 mAP and 69.5 mAOS under 5-scan aggregation.

#### 4.4.2. Effect of Radar Point Cloud Density

We investigate how radar point cloud density—controlled by the interpolation parameter *k*—affects detection. A positive *k* indicates that *k* points are randomly interpolated per 3D ROI, while a negative *k* means that *k* points are randomly removed. As reported in Table 4, sparser point clouds degrade performance, whereas increasing the quantity of interpolated points yields consistent gains in both mAP and mAOS. The optimal trade-off is achieved at k=12, where the model attains its peak mAP of 69.3 and mAOS of 59.8; beyond this value (k=15), performance slightly declines, indicating that excessive interpolation introduces noise.

#### 4.4.3. Effect of 3D ROI Quality

**Figure 10:**
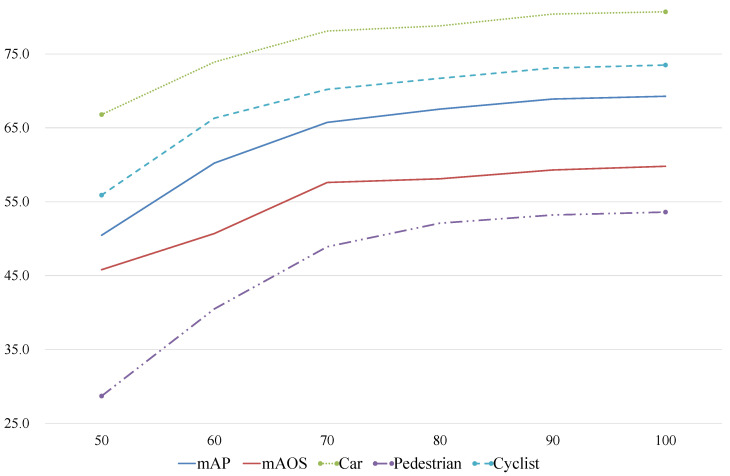
As the number of ROI-generator training epochs increases, the mAP and mAOS performance gradually improves.

This ablation assesses the impact of 3D ROI quality on final detection accuracy. We extract checkpoints from different training epochs of the ROI generator and evaluate each checkpoint’s downstream effect on 3D detection. As shown in Table 5, the strongest results in this ablation (mAP: 69.3, mAOS: 59.8) are obtained after 100 epochs of heatmap generator training. Figure 10 further illustrates that improving ROI quality leads to consistent gains in per-category AP as well as overall mAP and mAOS. These findings confirm that high-quality ROIs enable more precise point cloud densification in object regions, thereby improving the discriminability between object and background points and boosting detection accuracy.

#### 4.4.4. Effect of Transformation-Invariant Feature

We analyze the individual and combined contributions of transformation-invariant velocity encoding and heatmap features. For the node representation, the radial velocity $\left(v_{r},v_{c}\right)$ in VOD is replaced by the normalized Cartesian components $\left(v_{x},v_{y},v_{z}\right)$ aligned with the coordinate axes. As reported in Table 6, using the transformation-invariant encoding alone raises mAP to 62.7. Adding the heatmap feature alone yields 64.2 mAP. The full model, which combines both strategies, achieves a final mAP of 69.3 alongside an mAOS of 59.8, confirming that the two components provide complementary benefits.

#### 4.4.5. Effect of Camera-Derived Prior in SAA

A legitimate concern is whether the SAA module’s performance gain stems primarily from the camera detector (i.e., the ROI quality) rather than from genuine radar-camera fusion. To investigate this, we conduct the following controlled comparisons on the VOD dataset: (i) *Camera ROI only*: we use the image-derived 3D ROI bounding boxes directly as detection results without any radar processing—this isolates the contribution of the camera detector alone; (ii) *Heatmap only*: we feed only the heatmap features to the HyperGCN without any radar point cloud, testing whether the spatial probability map alone suffices for detection; (iii) *Pseudo-points without real radar*: we generate pseudo-points using SAA but deliberately exclude all real radar returns, retaining only the interpolated points—this reveals whether the model relies on the camera prior or on the physical radar measurements. The results are summarized in Table 7.

The “Camera ROI only” setting yields an mAP of 25.3, far below the full model’s 69.3, confirming that the camera detector alone is insufficient for accurate 3D detection. The “Heatmap only” setting achieves 31.7 mAP, indicating that the spatial prior provides useful but limited information. The “Pseudo-points without real radar” setting reaches 42.1 mAP, substantially lower than the full model (69.3), demonstrating that while pseudo-points contribute meaningful densification, the real radar returns (especially their velocity and rcs attributes) are indispensable for high-fidelity detection. These results collectively confirm that the performance gain arises from the synergistic fusion of camera guidance and radar measurements, not from the camera detector alone.

#### 4.4.6. Physical Plausibility of Pseudo-Point Generation

The SAA module generates pseudo-points by random interpolation within 3D ROIs and assigns their $\left(rcs,v_{r},v_{c},t\right)$ attributes via KNN averaging from neighboring real radar returns. We acknowledge that this approximation does not capture the full physical complexity of real radar measurements, where rcs depends on target material, surface geometry, and incident angle, and where velocity is determined by the target’s motion state. However, we argue that the KNN-based assignment is a reasonable first-order approximation for the following reasons: (i) objects within the same ROI typically share similar material properties (e.g., a car body is predominantly metal), so rcs values of neighboring points provide a representative estimate; (ii) the radial velocity $v_{r}$ of points within the same object is highly correlated because the object moves as a rigid body, making the KNN average a physically sound estimate; (iii) the timestamp *t* is a frame-level constant shared by all points, so its assignment is exact. To quantify the sensitivity to the pseudo-feature generation strategy, we compared KNN averaging with two alternatives: zero-filling (setting pseudo-point features to zero) and random sampling (drawing from the distribution of real point features). KNN averaging achieves 69.3 mAP, compared to 64.8 for zero-filling and 66.1 for random sampling, confirming that KNN provides the most physically consistent estimate among the tested strategies.

## 5. Discussion

In this section, we critically analyze the proposed method in the broader context of multi-modal perception, discuss the physical phenomena that affect sensor performance, and delineate the limitations of the current approach.

### 5.1. Comparison with Alternative Fusion Paradigms

The proposed graph-based fusion differs fundamentally from probabilistic matching approaches such as Bayesian inference networks. In a Bayesian formulation, the association between radar detections and camera observations is treated as a latent variable, and posterior probabilities are computed via likelihood functions that model sensor-specific noise characteristics. While this framework offers principled uncertainty quantification, it requires explicit modeling of measurement likelihoods—a non-trivial task for 4D radar due to the complex relationship between target properties and radar returns. Our approach, by contrast, learns the cross-modal associations implicitly through hypergraph construction and graph convolution, sidestepping the need for hand-crafted likelihood models. The trade-off is reduced interpretability: the graph-based method does not provide explicit posterior probabilities for data association. A hybrid approach that uses Bayesian inference for initial radar-camera association and graph neural networks for relational reasoning could combine the strengths of both paradigms and represents a promising direction for future work.

### 5.2. Physical Phenomena Affecting Sensor Performance

Several physical phenomena impact the performance of both radar and camera modalities in our system. For radar, multipath propagation occurs when radar signals reflect off multiple surfaces before returning to the receiver, producing ghost detections at incorrect ranges. This effect is particularly pronounced in urban canyons with reflective building facades. Radome interference and mutual interference between multiple radar-equipped vehicles can also corrupt measurements. The sparse nature of 4D radar point clouds means that even a few multipath artifacts can significantly distort the constructed hypergraph. For cameras, optical aberrations—including lens distortion, chromatic aberration, and vignetting—degrade image quality and propagate errors into the 2D detection and heatmap generation stages. Occlusions by other vehicles, infrastructure, or weather effects (raindrops on the lens, fog) reduce the effective field of view and can cause incomplete ROI proposals. Our current system does not explicitly model these phenomena; however, the transformation-invariant encoding provides partial robustness to geometric distortions, and the hypergraph structure can tolerate missing nodes (i.e., occluded objects) as long as sufficient radar returns are available. Future work could incorporate explicit multipath mitigation and optical aberration correction as preprocessing steps.

### 5.3. Limitations and Future Directions

The current approach has several notable limitations. First, the SAA densification is confined to camera-visible regions, meaning that objects outside the camera field of view receive no augmentation—a constraint that could be partially addressed by incorporating surround-view cameras. Second, the KNN-based pseudo-feature assignment is a first-order approximation that does not model the dependency of rcs on incident angle or target pose; a learned feature generator conditioned on image appearance could provide more physically accurate pseudo-features. Third, the fixed interpolation parameter *k* does not adapt to object size or distance; an adaptive densification strategy that adjusts *k* based on ROI dimensions could improve efficiency. Finally, the nuScenes results demonstrate that the architecture’s performance is bounded by the quality of the radar sensor: the absence of elevation in 3D radar fundamentally limits the 3D detection capability, regardless of the fusion strategy employed.

## 6. Conclusions

We have introduced a multi-modal radar-camera perception system built on graph-based relational reasoning for high-fidelity 3D scene understanding in autonomous navigation. The architecture unifies four interdependent processing stages—image-guided radar densification (SAA), pose-invariant cross-modal integration, hypergraph-based relational modeling, and HyperGCN inference—into a cohesive end-to-end pipeline that tackles the low-density limitation of radar point clouds at its root. Leveraging both the velocity-rich temporal cues of radar and the visually detailed appearance provided by cameras, the system achieves competitive performance among the radar-centric baselines considered in this study, with evaluation scores of 69.3 mAP and 59.8 mAOS recorded on the VOD benchmark. Comprehensive ablation experiments confirm that pose-invariant encoding and heatmap-guided feature fusion deliver independent and mutually reinforcing improvements.

Looking ahead, we plan to investigate adaptive parameter scheduling to improve robustness under complex and varying scenarios, as well as dynamically adjustable densification strategies that can balance point cloud density against sensing range. These extensions are expected to mitigate the limitations of fixed-parameter interpolation and further elevate perception performance in real-world deployment.

## Figures and Tables

**Table 1:** VOD benchmark results. The official evaluation metrics are mAP and mAOS. Dense LiDAR-only models provide the strongest upper-bound results, while the proposed radar–camera setting improves over the radar-only baselines considered here.

Method	Modality	Features	mAP	mAOS	Car	Ped.	Cyclist
MonoRCNN++ [45]	Camera	R,G,B	17.6	10.2	30.7	13.2	8.9
IA-SSD [46]	LiDAR	x,y,z,intensity	85.5	76.3	93.6	77.2	85.7
	Radar	x,y,z,rcs	55.1	45.9	72.1	33.6	59.7
	Radar	$x,y,z,rcs,v_{r}$	66.2	56.3	82.4	45.2	71.1
PointPillars [19]	LiDAR	x,y,z,intensity	81.6	70.3	90.8	71.4	82.5
	Radar	x,y,z,rcs	52.3	41.0	67.3	31.0	58.7
	Radar	$x,y,z,rcs,v_{r}$	63.0	56.8	74.1	47.8	67.1
PointRCNN [20]	LiDAR	x,y,z,intensity	83.2	71.8	92.5	73.6	83.6
	Radar	x,y,z,rcs	53.7	42.3	68.5	33.1	59.6
	Radar	$x,y,z,rcs,v_{r}$	64.6	58.1	75.9	48.6	69.3
Ours	Radar+Camera	rcs,t,heatmap	60.2	48.3	70.5	46.2	63.9
Ours	Radar+Camera	$\vec{V},rcs,t,heatmap$	**69.3**	**59.8**	**80.7**	**53.6**	**73.5**

**Table 2:** nuScenes benchmark results. The performance bound (P.B.) is calculated based on the ratio of annotated objects that produce radar detections. Class metrics with AP below 1% are excluded. “R” and “C” indicate the radar and camera modality, respectively. “–” implies the item was not available. Bold indicates the strongest result among the listed methods. Abbreviations: Modality (Mod.), Pedestrian (Ped.).

	Mod.	Classes	AP	ATE	AOE	ASE	AVE
Ours	R + C	Car	**13.2**	**0.66**	**0.37**	**0.19**	**0.93**
		Truck	**5.6**	**0.76**	**0.48**	**0.32**	**1.86**
R-PointGNN [47]	R	Car	10.1	0.69	0.38	0.2	0.95
PointNet [13]	R	Car	5.2	1.11	0.72	0.2	1.16
P.B.		Car	58.0	–	0.01	–	–
		Ped.	21.0	–	0.13	–	–
		Barrier	29.0	–	0.14	–	–
		Truck	69.0	–	0.29	–	–

**Table 3:** Multi-scan aggregation results. The 3-scans and 5-scans denote the last three and five scan frames, respectively. Our method achieves better performance on five radar scans (mAP: 73.9, mAOS: 69.5). Abbreviations: Pedestrian (Ped.).

Method	3-Scans	5-Scans	mAP	mAOS	Car	Ped.	Cyclist
PointPillars			63.0	56.8	74.1	47.8	67.1
PointPillars	✔		69.4	66.2	77.3	55.1	75.8
PointPillars		✔	70.8	67.3	77.8	58.2	76.5
Ours			69.3	59.8	80.7	53.6	73.5
Ours	✔		72.5	68.2	81.2	60.3	76.1
Ours		✔	**73.9**	**69.5**	**82.4**	**61.5**	**77.8**

**Table 4:** Effect of interpolation parameter *k* on detection performance. Bold face highlights the optimal performance level.

K-Points	mAP	mAOS	Car	Pedestrian	Cyclist
k=−6	59.1	46.7	69.4	44.8	63.2
k=−3	61.9	51.7	72.9	46.2	66.5
k=0	64.9	55.6	76.8	48.9	69.1
k=6	66.7	57.2	78.1	51.1	70.9
k=9	67.9	58.6	79.2	52.3	72.2
k=12	**69.3**	**59.8**	**80.7**	**53.6**	**73.5**
k=15	68.6	59.3	80.2	52.9	72.8

**Table 5:** Effect of heatmap generator training epochs on detection performance. The strongest result in this ablation is obtained at Epochs=100.

Epochs	mAP	mAOS	Car	Pedestrian	Cyclist
50	50.5	45.8	66.8	28.7	55.9
60	60.2	50.7	73.9	40.5	66.3
70	65.7	57.6	78.1	48.9	70.2
80	67.5	58.1	78.8	52.1	71.7
90	68.9	59.3	80.4	53.2	73.1
100	**69.3**	**59.8**	**80.7**	**53.6**	**73.5**

**Table 6:** Ablation of pose-invariant encoding and heatmap features. Models combining both strategies perform better than either individual strategy in this ablation. Abbreviations: Pose-invariant encoding (Pose-Inv.), Pedestrian (Ped.).

Pose-Inv.	Heatmap	mAP	mAOS	Car	Ped.	Cyclist
✔		62.7	54.2	73.6	46.9	67.5
	✔	64.2	54.9	75.8	48.5	68.3
✔	✔	**69.3**	**59.8**	**80.7**	**53.6**	**73.5**

**Table 7:** Ablation study on the contribution of camera-derived prior vs. radar features in SAA. The full model (real radar + pseudo-points + heatmap) significantly outperforms all reduced variants, confirming that the gains stem from genuine radar-camera fusion rather than camera prior alone.

Setting	mAP	mAOS	Car	Ped.	Cyclist
Camera ROI only	25.3	18.6	40.1	15.2	20.7
Heatmap only	31.7	22.4	48.3	19.8	26.9
Pseudo-pts w/o real radar	42.1	33.8	56.7	28.4	41.2
Full model (Ours)	**69.3**	**59.8**	**80.7**	**53.6**	**73.5**

## Data Availability

The source code and tools are available at https://github.com/denyz/GEN (accessed on 13 July 2026).

## Abbreviations

The following abbreviations are used in this manuscript:

MMW	Millimeter-Wave
SAA	Supervised Attention Augmentation
GCN	Graph Convolutional Network
HyperGCN	Hypergraph Convolutional Network
BBox	Bounding Box
ROI	Region of Interest
BEV	Bird’s Eye View
MLP	Multi-Layer Perceptron
FPN	Feature Pyramid Network
NMS	Non-Maximum Suppression
mAP	Mean Average Precision
mAOS	Mean Average Orientation Similarity
VOD	View-of-Delft
AP	Average Precision
ATE	Average Translation Error
AOE	Average Orientation Error
ASE	Average Scale Error
AVE	Average Velocity Error
